# Unravelling *var* complexity: Relationship between DBLα types and *var* genes in *Plasmodium falciparum*

**DOI:** 10.3389/fpara.2022.1006341

**Published:** 2023-01-09

**Authors:** Mun Hua Tan, Heejung Shim, Yao-ban Chan, Karen P. Day

**Affiliations:** ^1^ Department of Microbiology and Immunology, The University of Melbourne, Bio21 Institute, Melbourne, VIC, Australia; ^2^ School of Mathematics and Statistics/Melbourne Integrative Genomics, The University of Melbourne, Melbourne, VIC, Australia

**Keywords:** antigenic variation, DBLα tag, recombination, Africa, Asia, ups, PfEMP1

## Abstract

The enormous diversity and complexity of *var* genes that diversify rapidly by recombination has led to the exclusion of assembly of these genes from major genome initiatives (e.g., Pf6). A scalable solution in epidemiological surveillance of *var* genes is to use a small ‘tag’ region encoding the immunogenic DBLα domain as a marker to estimate *var* diversity. As *var* genes diversify by recombination, it is not clear the extent to which the same tag can appear in multiple *var* genes. This relationship between marker and gene has not been investigated in natural populations. Analyses of *in vitro* recombination within and between *var* genes have suggested that this relationship would not be exclusive. Using a dataset of publicly-available assembled *var* sequences, we test this hypothesis by studying DBLα-*var* relationships for four study sites in four countries: Pursat (Cambodia) and Mae Sot (Thailand), representing low malaria transmission, and Navrongo (Ghana) and Chikwawa (Malawi), representing high malaria transmission. In all study sites, DBLα-*var* relationships were shown to be predominantly 1-to-1, followed by a second largest proportion of 1-to-2 DBLα-*var* relationships. This finding indicates that DBLα tags can be used to estimate not just DBLα diversity but *var* gene diversity when applied in a local endemic area. Epidemiological applications of this result are discussed.

## 1 Introduction

The microbiological paradigm for surveillance of diverse pathogens requires documenting the diversity of genes encoding major variant surface antigens [e.g (World Health Organization; World Health Organization; Nicholson et al., 2003; Wang et al., 2021)]. In the case of the malaria parasite, *Plasmodium falciparum*, the major surface antigen of the blood stages is known as *P. falciparum* erythrocyte membrane-1 protein (PfEMP1) encoded by the *var* multigene family. This molecule plays a key role in the biology and epidemiology of *P. falciparum*. Differential switching of *var* genes results in immune evasion by clonal antigenic variation (Smith et al., 1995; Su et al., 1995). Furthermore, PfEMP1 mediates sequestration *via* cytoadherence (Baruch, 1999; Newbold et al., 1999; Chen et al., 2000) to allow parasite maturation and replication. The molecule is also considered a virulence factor as cytoadhesion characteristics of specific variants are associated with the incidence of severe disease (Wang et al., 2012; Bengtsson et al., 2013; Turner et al., 2013; Bernabeu et al., 2016; Magallón-Tejada et al., 2016; Lennartz et al., 2017; Tonkin-Hill et al., 2018; Tessema et al., 2019). Unlike merozoite surface antigens that are exposed to the immune system for seconds to minutes, PfEMP1 variants remain on the surface for up to 24 hours, making these variants subject to intense immune selection.

*P. falciparum* has approximately 40 to 60 distinct *var* genes in the haploid genome. They are distributed across all 14 chromosomes but are mostly located within subtelomeric regions, with a small subset clustered in the central regions of specific chromosomes (Gardner et al., 2002; Otto et al., 2018). Analysis of *var* genes in seven *P. falciparum* genomes has shown that *var* repertoires and genes evolve by recombination (Rask et al., 2010). Subtelomeric regions, which contain *var* genes, have shown susceptibility to high rates of ectopic recombination during both meiosis and mitosis, based on observations largely from *in vitro* long-term cultured lines (Freitas-Junior et al., 2000; Duffy et al., 2009; Bopp et al., 2013; Claessens et al., 2014; Zhang et al., 2019). The proposed combined mechanisms involving telomere healing and homologous recombination (HR) for DNA repair (Calhoun et al., 2017; Zhang et al., 2019) place a limit on possible recombination events and thus create chimeric *var* genes that are intact and functional (Bopp et al., 2013; Claessens et al., 2014; Zhang et al., 2019). These constraints are reflected in the higher frequency of observed recombination between *var* genes classified in the same ups group (Bopp et al., 2013; Claessens et al., 2014; Feng et al., 2022) where these groupings define chromosomal locations. Recombination has also been shown *in vitro* to preferentially occur between sequence pairs belonging to the same domain class (Smith et al., 2000; Kraemer and Smith, 2006; Rask et al., 2010), though this constraint does not appear to apply to recombination between domain subclasses (Claessens et al., 2014).

Assessing *var* diversity in endemic areas is not a simple task. Many *var* genes in different parasite genomes are non-orthologous and the overall numbers of *var* genes in a genome vary in different *P. falciparum* isolates (Otto et al., 2018). Multigenome infections (i.e., complex infections) are also common in individuals. These factors, in addition to the often uneven sequence coverage of *var* genes during whole genome sequencing, greatly challenges the sequence assembly process. Until recently (Dara et al., 2017; Otto et al., 2019; Mackenzie et al., 2022), no large repository of *var* sequences existed as these genes were routinely excluded from studies using whole genome sequencing datasets (e.g., Pf3k, Pf6). The assembled *var* dataset made publicly available by Otto et al. (2019) therefore represents the largest *var* repository currently available. Sampled from 2,459 clinical isolates from 15 countries (six in Asia, nine in Africa), the 377,924 *var* sequences from the ‘Full Dataset’, however, contains a mix of complete and partial *var* sequences, further highlighting the bioinformatic challenges in recovering full length *var* genes.

Of all domains encoded by *var*, the DBLα domain has been identified as the domain with the highest recombination rate *in vitro* (Claessens et al., 2014) and is immunogenic with variant-specific epitopes, recognized serologically in an age-dependent manner (Barry et al., 2011). Thus, a scalable solution to studying this highly-diverse *var* multigene family in natural populations has been to target a small conserved region within the DBLα domain of *var* genes (i.e., DBLα tags) (Barry et al., 2007; Chen et al., 2011; Yalcindag et al., 2012; Tessema et al., 2015; Day et al., 2017; Rougeron et al., 2017; Ruybal-Pesántez et al., 2017). This marker has been used as a proxy to characterize *var* diversity (Barry et al., 2007; Chen et al., 2011; Albrecht et al., 2006, 2010) and repertoire structure (Day et al., 2017), and has served as empirical data for the analysis of interventions (Tiedje et al., 2022; Pilosof et al., 2019). Studies in high transmission showed limited overlap of *var* DBLα repertoires (Day et al., 2017; Ruybal-Pesántez et al., 2017; Tiedje et al., 2017; Ruybal-Pesántez et al., 2022) as a consequence of immune selection (He et al., 2021). Subsequently, these studies have also served as proof of concept for the use of DBLα tags for estimating complexity/multiplicity of infection (MOI) in surveillance (Ruybal-Pesántez et al., 2022; Tiedje et al., 2022) and this has been evaluated compared to SNP barcoding using a mathematical modelling exercise (Labbé et al., 2023). These studies made the assumption that each DBLα tag represents a unique *var* gene, especially for the non-upsA *var*. If the high mitotic recombination rates observed by Claessens et al., 2014 *in vitro* also occur *in vivo*, this assumption would be incorrect in natural populations as we would see many chimeric *var* genes that share a same DBLα tag. Now that a dataset of *var* gene sequences is recently available (Otto et al., 2019), we can test this assumption to better understand the DBLα-*var* relationship (i.e., whether it is a specific 1-to-1 or 1-to-many).

Using a subset of the assembled *var* dataset published by Otto et al. (2019), we investigated the relationships between DBLα tags and *var* in four study sites located in four countries: Pursat (Cambodia) and Mae Sot (Thailand), representing low malaria transmission, and Navrongo (Ghana) and Chikwawa (Malawi), representing high malaria transmission. In all four study sites, DBLα-*var* relationships were shown to be predominantly specific (i.e., 1-to-1) with much smaller proportions of DBLα types associated with many *var* exon 1 sequences. We attribute this specificity to the field observation that most DBLα sequences are found to be rare in a local population and that targeting sequences encoding a fast-evolving domain will yield a highly-specific domain-to-*var* relationship. Finding mostly highly-specific DBLα types has an important and practical implication; it indicates that the diversity of DBLα types can be used as an approximation of diversity levels of *var* genes (more specifically, *var* exon 1) in genomic epidemiological studies conducted in a local endemic area, supporting a simple strategy to monitor and tackle the complexity of the *var* system in endemic areas where whole genome sequencing efforts often ignore these important genes.

## 2 Methods

### 2.1 Data selection

**Figure 1:**
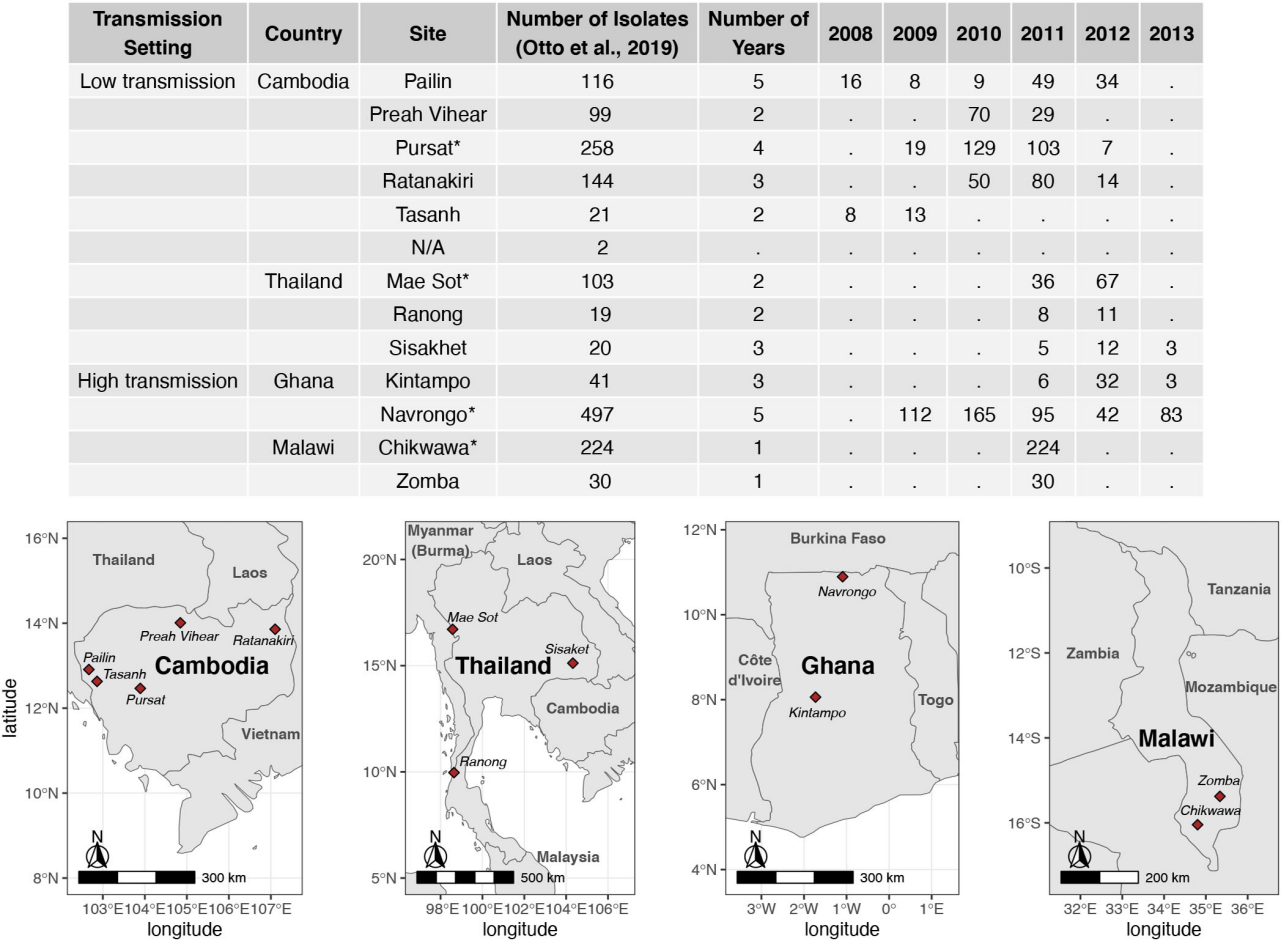
Number of isolates with available *var* sequences from study sites in Cambodia, Thailand, Ghana, and Malawi. *Var* sequences were downloaded from the published ‘Full Dataset’ by Otto et al. 2019. Study sites marked with ‘*’ in the table were selected for analysis in this study.

Assembled *var* sequences are available for 15 countries in the ‘Full Dataset’ published in Otto et al. (2019), of which six are in Asia and nine are in Africa (**Figure S1** in **Data Sheet 1**), and represent clinical isolates with varying malaria disease status (e.g., severe, acute/uncomplicated) (Consortium P., 2016; MalariaGen et al., 2021). The four countries (Cambodia, Thailand, Ghana, Malawi) with the highest number of isolates were identified. For each country, the study site with the highest number of isolates available was selected for analysis in this study. These are Pursat (Cambodia) and Mae Sot (Thailand), representing study sites with low malaria transmission, and Navrongo (Ghana) and Chikwawa (Malawi), representing study sites with high malaria transmission (**Figure 1**). Maps in **Figure 1** were produced using data from the *rnaturalearth* R package (South, 2017) and visualized with *ggplot2* (Wickham, 2016).

### 2.2 Extraction of complete *var* exon 1 sequences

**Figure 2:**
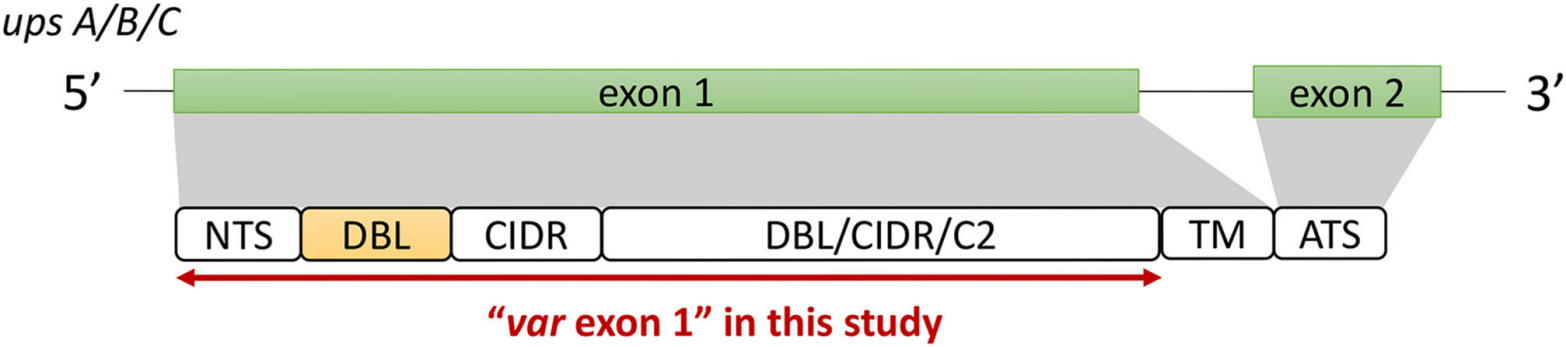
General *var* gene structure and "*var* exon 1" in this study (see Methods for details). Generally, the *var* gene structure consists of two exons; a larger exon 1 that encodes the extracellular antigenic portion of the PfEMP1 protein that is ‘exposed’ to host receptors and antibodies, and a smaller, semi-conserved exon 2 that remains intracellular (Su et al., 1995; Smith et al., 2000). The first exon typically consists of an N-terminal segment (NTS) on the 5’ end, followed by sequences encoding multiple semi-conserved domains such as the Duffy binding-like domains (DBL), cysteine-rich interdomain regions (CIDR) and/or C2 domains that, when combined, make up *var*ious *var* domain compositions and structures (Kraemer and Smith, 2006; Rask et al., 2010). While domains such as the DBLδ and CIDRα have been shown to be highly diverse (Otto et al., 2019), the DBLα domain has the added importance of being present in most *var* genes (Rask et al., 2010). Based on the upstream promoter sequence, *var* genes in this multigene family can be further divided into four subgroups of upsA, upsB, upsC, and upsE, with each group exhibiting unique characteristics related to general genomic location, transcription direction, host receptor binding, and disease severity (Gardner et al., 2002; Lavstsen et al., 2003; Kraemer and Smith, 2006).

The ‘Full Dataset’ was filtered to retain only complete *var* exon 1 sequences. The majority of *var* genes have been reported to contain an N-terminal segment (NTS) on the 5’ end and a transmembrane region (TM) on the 3’ end of exon 1 (Rask et al., 2010). Therefore, in this study, the NTS and TM domains were used to represent the left (5’ end) and right (3’ end) boundaries of *var* exon 1, respectively (**Figure 2**). Given that this study aims to investigate relationships between DBLα types and *var* sequences, *var* sequences with missing DBLα domain annotations were excluded, and in doing so, *var2csa* genes that do not contain the DBLα domain and *var3* genes that contain a particularly distinct DBLα-ζ hybrid domain were also not analyzed. In summary, to be considered as containing a complete *var* exon 1, the following criteria must be met:

- The *var* sequence must be translatable and must begin with a start codon (‘Met’)
- The *var* sequence must not contain gaps (‘N’s)
- The *var* sequence must encode exactly one NTS domain, exactly one DBLα domain and exactly one TM domain

To detect the presence of these domains, the annotation of domains provided by Otto et al. (2019) was firstly used to check that a sequence encodes both the NTS and DBLα domains. To further verify that a *var* encodes the NTS and TM domains, each sequence was translated according to its best reading frames and, using *hmmsearch* (Eddy, 2011), was searched for homology blocks HB20 (found in NTS) and HB21 (found in TM) (Rask et al., 2010). Domain score cut-offs of 10 and 20 were used for NTS and TM alignments, respectively, and these were determined from distributions of domain scores from alignments of *var* genes recovered from a set of 16 whole genome assemblies (**Figure S2** in **Data Sheet 1**), downloaded from PlasmoDB and NCBI (14 from PlasmoDB (Otto et al., 2018), two from NCBI). *Var* sequences that did not contain alignments to both homology blocks were removed. *Var* sequences containing multiple NTS, DBLα or TM domains were also removed as these may be a result of mis-assemblies. The remaining sequences were then truncated on the 3’ end to include sequences from the start of the *var* gene to just before the region encoding the TM domain (i.e., excluding the TM sequence region) (**Figure 2**). This was done using the coordinate position of the alignment to HB21 (i.e., ‘from’ position (*env coords*)). *Var1* sequences were also identified based on information in the sequence headers (Otto et al., 2019) and excluded.

### 2.3 Extraction of DBLα tags from *var* exon 1

Nucleotide sequences encoding the DBLα domains were extracted from complete *var* exon 1 sequences using domain annotations provided alongside the assembled *var* datasets (Otto et al., 2019). These DBLα sequences were further translated according to their best reading frame and, using *hmmsearch* (Eddy, 2011), the resulting amino acid sequences were further searched against positions 189 to 430 of the PFAM profile alignment (PF05424_seed.txt) to extract the ‘tag’ region. A domain score cut-off of 60 was used. This was determined from a distribution of domain scores from alignments of DBLα tags of *var* genes from a set of 16 whole genome assemblies (**Figure S2** in **Data Sheet 1**), downloaded from PlasmoDB and NCBI (as above). For each sequence, if 100 (out of 111) positions of the HMM profile were aligned (*hmm coords*), the best aligned hit was retained. The identified ‘from’ and ‘to’ positions (*env coords*) were used to extract the DBLα tag region that would have been typically amplified with universal degenerate primers (Taylor et al., 2000).

### 2.4 Clustering of DBLα tags into DBLα types

DBLα tags were clustered with *vsearch* (Rognes et al., 2016) using the *cluster_fast* function at a range of thresholds from 90% to 100% nucleotide identity, calculated over whole alignment lengths, including terminal gaps (*–iddef* 1). The 96% nucleotide identity threshold was selected for further downstream analyses to reflect the application of this method in the field and in current bioinformatic workflows when defining representative DBLα types [e.g (Barry et al., 2007; Ruybal-Pesántez et al., 2017; Tonkin-Hill et al., 2021)].

### 2.5 Sampling curves

Rarefaction curves for *var* exon 1 and DBLα types clustered at a threshold of 96% nucleotide identity were generated with the *rarecurve* function in the *vegan* R package (Oksanen et al., 2022) and plot with *ggplot2* (Wickham, 2016).

### 2.6 Classification of DBLα types and *var* into upsA or non-upsA groups

The *classifyDBLalpha* pipeline (Ruybal-Pesántez et al., 2017) was used to assign domain classes and subclasses to DBLα types. DBLα types that were assigned the DBLα1 domain class were classified into the upsA group whereas DBLα types assigned with the DBLα0 or DBLα2 domain classes were classified into the non-upsA group (i.e., upsB/upsC) (Rask et al., 2010). Similarly, *var* exon 1 sequences were also classified into the different ups groups based on the classification of their associated DBLα types.

### 2.7 Determination of DBLα-*var* relationships

**Figure 3:**
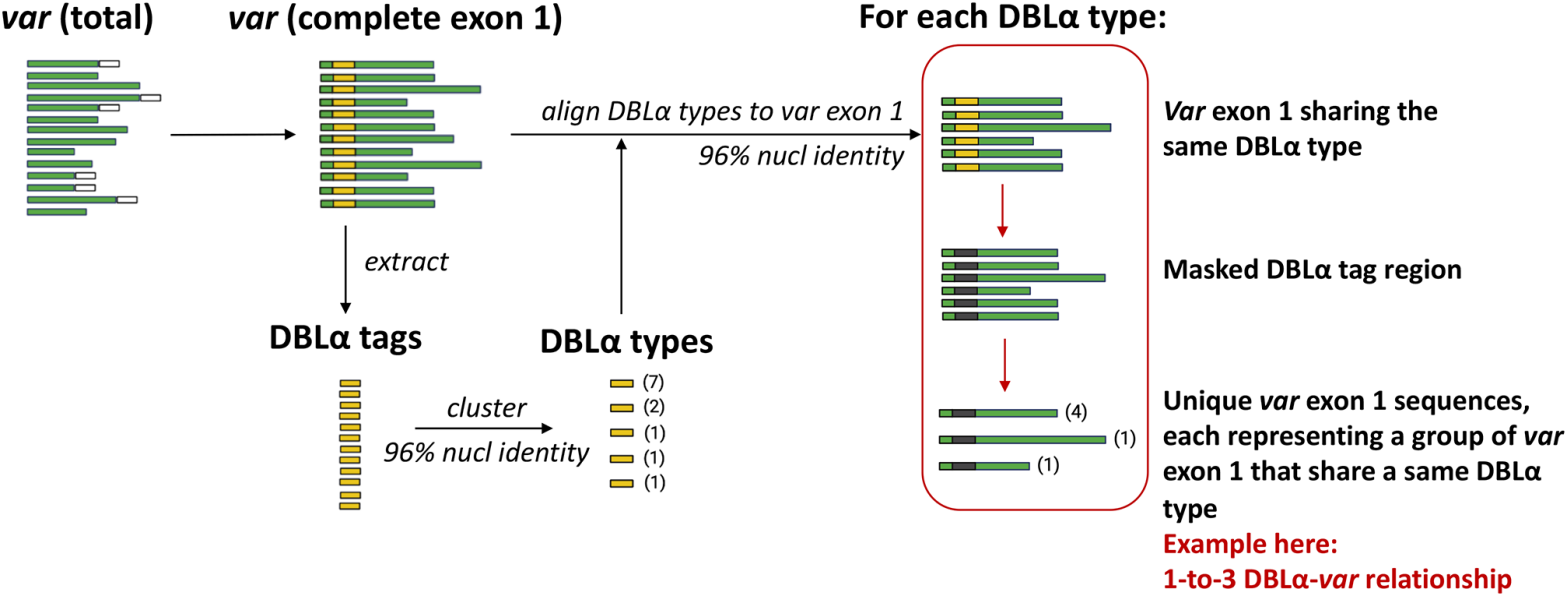
Overview of workflow to determine DBLα-var relationships. Numbers in parentheses represent the frequency of sequences.

An overview of the workflow to determine DBLα-*var* relationships is illustrated in **Figure 3**. DBLα types were globally aligned to *var* exon 1 with *vsearch* (Rognes et al., 2016) using the *usearch_global* function, according to the following configurations:

- Alignments of DBLα types to site-specific *var* (e.g., Pursat DBLα types to Pursat *var* exon 1)
- Alignments of DBLα types to country-specific *var* (e.g., Pursat DBLα types to Cambodia *var* exon 1)
- Alignments of DBLα types to continent-specific *var* (e.g., Pursat DBLα types to Asia *var* exon 1; Navrongo DBLα types to Africa *var* exon 1)
- Alignments of DBLα types to time-specific *var* (e.g., Pursat DBLα types to Pursat *var* exon 1 found in 2010)

DBLα types generated from clustering at a 96% nucleotide identity threshold were aligned to *var* exon 1 sequences at the same alignment threshold of 96%. Pairwise nucleotide identities of these alignments were calculated over the alignment length, excluding terminal gaps (*–iddef* 2). For each group of *var* exon 1 sequences that share a same DBLα type, the DBLα tag region of these *var* exon 1 sequences were masked with ‘N’s and dereplicated. The relationship between a DBLα type and *var* exon 1 was then determined from the number of unique *var* exon 1 sequences in each group of *var* exon 1 that share a same DBLα type. Therefore:

- a 1-to-1 relationship is defined as a DBLα type found in only one unique *var* exon 1
- a 1-to-2 relationship is defined as a DBLα type shared by two unique *var* exon 1
- a 1-to-*n* relationship is defined as a DBLα type shared by *n* unique *var* exon 1

### 2.8 Determination of DBLα type-to-domain relationships

From the same set of *var* exon 1 sequences as described above, sequences encoding the DBLα domain were extracted based on the annotation of domains provided by Otto et al. (2019). Similar to the method used to determine DBLα-*var* relationships, DBLα types were aligned to DBLα domain sequences at an alignment threshold of 96%. The DBLα type-to-domain relationship was then determined from the number of unique sequences encoding the DBLα domain in each group that share a same DBLα type.

### 2.9 Pairwise alignment of *var* exon 1

For each group of *var* exon 1 that share a same DBLα type, an all *vs* all sequence alignment of *var* exon 1 sequences in the group was performed using the *allpairs_global* option within *vsearch* (Rognes et al., 2016) and set to include all pairwise alignments (*–acceptall*). Pairwise nucleotide identities were estimated based on calculations over whole alignment lengths, including terminal gaps (*–iddef* 1), to account for differences in pairs of *var* exon 1 of variable lengths. Pairs of *var* exon 1 were randomly chosen as examples for alignments for different ranges of sequence similarity (40-50%, 50-60%, 60-70%, 70-80%, 80-90%, 90-99%, ≥99%), then aligned and visualized using mVISTA (Frazer et al., 2004) in glocal alignment mode (Shuffle-LAGAN) (conservation parameters: Min ID=70, Min Length=100) (Brudno et al., 2003). Domain organization of *var* genes were obtained from domain annotation information provided by Otto et al. (2019).

## 3 Results

### 3.1 Description of datasets representing low and high malaria transmission

**Figure 4:**
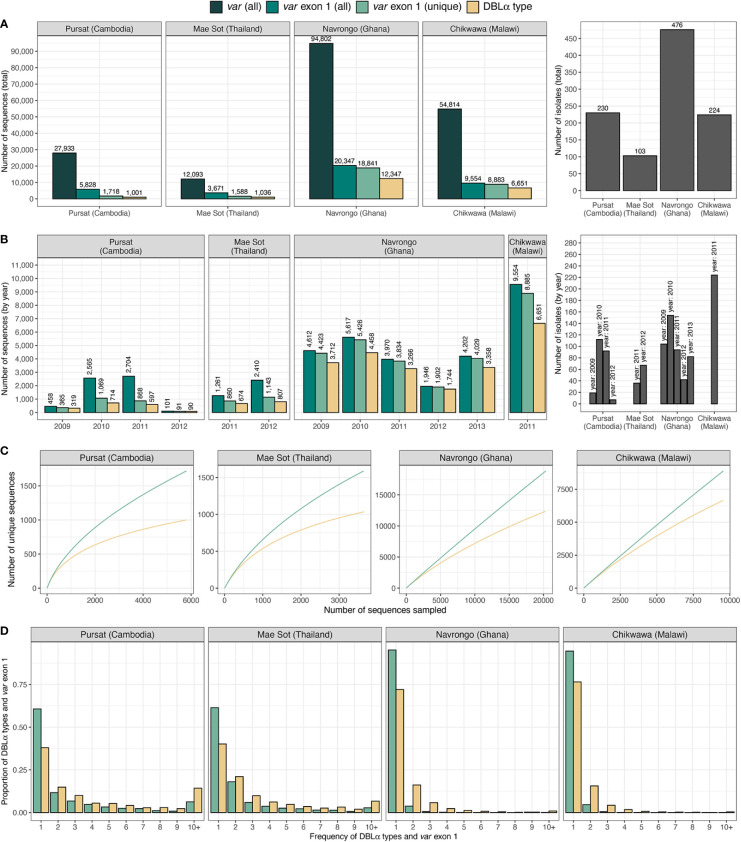
Description of *var* and DBLα type data from countries and sites. Number of sequences and isolates in the final dataset of *var* exon 1 (green) and DBLα type (yellow) sequences are presented **(A)** in total, and **(B)** by year. **(C)** Sampling curves of *var* exon 1 and DBLα type sequences. **(D)** Distribution of *var* exon 1 and DBLα type frequencies. Note: ‘*var* exon 1 (all)' represents a collection of all *var* genes inclusive of replicated sequences whereas ‘*var* exon 1 (unique)’ refers to representative *var* exon 1 sequences, each representing a group of *var* exon 1 that share a same DBLα type (clustered at 96%).

Datasets of clinical isolates from study sites in each of the countries in Asia (Pursat, Cambodia and Mae Sot, Thailand) and Africa (Navrongo, Ghana and Chikwawa, Malawi) were chosen as these have the largest number of isolates, to represent areas with low and high malaria transmission, respectively (**Figure 1**). An overview of the workflow used in this study is illustrated in **Figure 3**, with details and definitions outlined in Methods. In the ‘Full Dataset’ of assembled *var* sequences (Otto et al., 2019), more *var* sequences were available for African study sites (Navrongo: 94,802; Chikwawa: 54,814) compared to Asian study sites (Pursat: 27,933; Mae Sot: 12,093) (**Figure 4A**). Across all four study sites, an average of 22.6% of *var* sequences contained the complete *var* exon 1 sequence (**Figure 4A**, see **Figure 2** and Methods for a definition of ‘complete *var* exon 1’), with *var1* homologs excluded, due to their unusual characteristics as isolate-transcendent *var* genes that are semi-conserved and have unique features different from all other *var* genes (Kraemer et al., 2007). Homologs of other isolate-transcendent *var* genes (i.e., *var2csa* and *var3*) were also absent from these datasets; *var2csa* lacks a DBLα domain whereas *var3* contains a particularly distinct DBLα-ζ hybrid domain instead (Rask et al., 2010). The final cleaned datasets consisted of *var* exon 1 sequences from 230, 103, 476, and 224 isolates from Pursat, Mae Sot, Navrongo, and Chikwawa, respectively, sampled over varying time spans ranging from one to five years (**Figure 4B**). *Var* exon 1 and DBLα tag sequences are available as **Data Sheet 2**.

Using a previously-determined threshold of 96% nucleotide identity (Barry et al., 2007) that has been used in several studies [e.g (Chen et al., 2011; Day et al., 2017; Rougeron et al., 2017; Tessema et al., 2019)], clustering of DBLα tags at this threshold resulted in 1,001, 1,036, 12,347, and 6,651 DBLα types for Pursat, Mae Sot, Navrongo, and Chikwawa, respectively (**Figure 4**, results from varying threshold values shown in **Figure S3** in **Data Sheet 1**). Although larger numbers of *var* exon 1 and DBLα type sequences were available for Navrongo and Chikwawa, depths of sampling of both *var* exon 1 and DBLα type sequences were better achieved in Pursat and Mae Sot. Rarefaction sampling curves showed early indications of approaching data saturation in these study sites with low malaria transmission, more so for DBLα types than *var* exon 1 (**Figure 4C**). On the other hand, substantially elevated diversity of *var* exon 1 and DBLα types in high-transmission study sites was evident, with curves that continue to progressively increase with minimal signs of plateauing, indicative of under-sampling of both *var* exon 1 and DBLα type sequences in these datasets. When explored by ups groups, DBLα types were observed to most closely reflect diversity estimates of *var* exon 1 in non-upsA genes and in high-transmission sites (**Figure S4** in **Data Sheet 1**). In all study sites, differences in the number of DBLα types and *var* exon 1, as well as deflections in the curves, provide preliminary indications of some level of sharing of DBLα types between different *var* exon 1 sequences.

Regardless of transmission intensity, most *var* exon 1 and DBLα types were present in these datasets at low frequencies, with median frequencies of 1 or 2 at each study site (**Figure 4D**). However, within datasets for each study site, there were also smaller subsets of *var* exon 1 and DBLα types that were observed many times. The highest frequency *var* exon 1 in Pursat (PH0055-C.g29; NTS-DBLα-CIDRα-DBLδ-CIDRγ-DBLϵ-DBLϵ-DBLϵ), Mae Sot (PD0461-C.g223; NTS-DBLα-CIDRα-DBLδ-CIDRγ), Navrongo (PF0035-C.g504; NTS-DBLα-CIDRα-DBLδ-CIDRβ), and Chikwawa (PT0041-C.g416; NTS-DBLα-CIDRα-DBLδ-CIDRβ) were seen 83, 47, 39, and 20 times, respectively, in each population. The ‘NTS-DBLα-CIDRα-DBLδ-CIDRβ’ and ‘NTS-DBLα-CIDRα-DBLδ-CIDRγ’ domain architectures have been previously shown to be the most frequently observed in known *var* genes (Kraemer and Smith, 2006). On the other hand, the triple DBLϵ structure of the highest frequency *var* exon 1 in Pursat is less common; this *var* gene may potentially be defined as domain cassette 7 (DC7) in Rask et al. (2010). Notably, although the highest frequency *var* exon 1 in Navrongo and Chikwawa were of comparable lengths (5,226 and 5,184 nucleotides) and shared the same domain composition and structure, these sequences shared only 59.5% nucleotide identity and are, therefore, very different *var* genes.

### 3.2 Site-specific analysis suggests strong DBLα-*var* relationships in local populations

The workflow to determine varying degrees of relationships between DBLα types and *var* exon 1 is detailed in **Figure 3** and Methods. For simplicity and ease of understanding, we refer to these quantitative relationships between DBLα types and *var* exon 1 as “DBLα-*var* relationships”. For example, a 1-to-1 DBLα-*var* relationship refers to a DBLα type that is associated with only one unique *var* exon 1 sequence.

**Figure 5:**
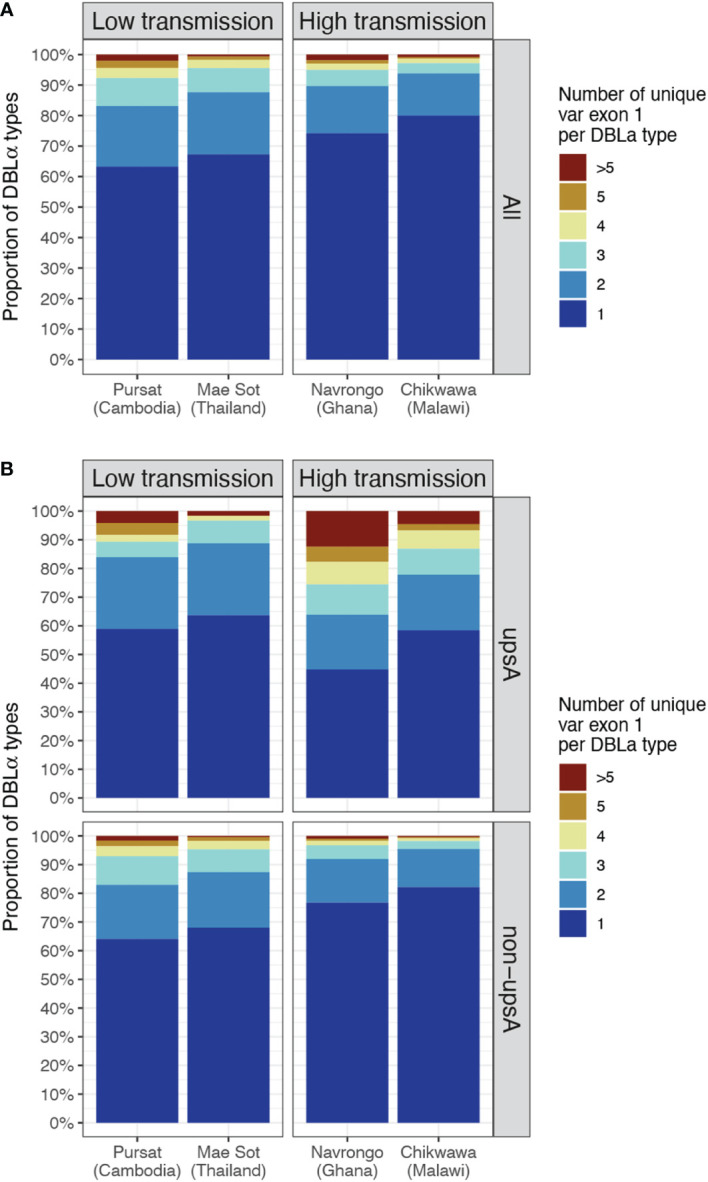
DBLα-*var* relationships based on alignments of DBLα types to site-specific *var* exon 1. The relationship between a DBLα type and *var* exon 1 is represented by the number of unique *var* exon 1 sequences that share a same DBLα type (clustered at 96% nucleotide identity threshold). Example: "1" represents "1-to-1", ">5" represents “1-to-many” DBLα-*var* relationships. **(A)** DBLα-*var* relationships for all DBLα types **(B)** DBLα-*var* relationships for DBLα types assigned to upsA and non-upsA groups based on DBLα domain classes. The minor upsA group accounts for an average of only 17.05% and 8.6% of DBLα types in low and high-transmission study sites, respectively.

In site-specific analysis (i.e., alignment of DBLα types to *var* exon 1 available from a local study site, see Methods), a consistent pattern of proportions of DBLα-*var* relationships was observed across all study sites (**Figure 5** and **Data Sheet 3**). For all four study sites, mainly 1-to-1 DBLα-*var* relationships were observed, indicating a highly-specific relationship between these DBLα types and *var* exon 1 sequences (**Figure 5A**, dark blue). On average, these 1-to-1 relationships were seen for 65.3% of DBLα types in low-transmission sites and 77.1% of DBLα types in high-transmission sites. This was followed by a second largest proportion of DBLα types linked to two different *var* exon 1 sequences (lighter blue, i.e., 1-to-2) and, finally, much smaller proportions of DBLα types linked to many *var* exon 1 sequences (red, i.e., 1-to-many or >5 unique *var* exon 1 per DBLα type). These relationships were also found to be robust for DBLα types clustered and aligned at varying thresholds, ranging from 90 to 100% nucleotide identities (**Figure S5** in **Data Sheet 1**). A further analysis to determine relationships between DBLα types and sequence regions encoding the complete DBLα domain (i.e., complete DBLα sequence) showed substantially higher levels of 1-to-1 and 1-to-2 specific type-to-domain relationships (**Figure S6** in **Data Sheet 1**), which implies that the observed 1-to-many DBLα-*var* relationships result from variation in sequence regions of the *var* gene outside of the DBLα domain. In the 1-to-1 DBLα-*var* relationship category, *var* exon 1 sequences associated with highly-specific DBLα types shared mostly 60 to 70% nucleotide sequence identity with other *var* exon 1 sequences and also varied considerably in sequence length (**Figure S7** in **Data Sheet 1**), suggesting that DBLα types in these highly-specific DBLα-*var* relationships also likely represent antigenically very different *var* genes; this remains to be explored and verified in future proteomic studies.

Classification of DBLα types into upsA and non-upsA (i.e., upsB/upsC) groups showed that, in all study sites, the majority of DBLα types were assigned to the non-upsA group (Pursat: 83.2%, Mae Sot: 82.7%, Navrongo: 92.0%, Chikwawa: 90.8%). All levels of DBLα-*var* relationships were observed for DBLα types in both upsA and non-upsA groups (**Figure 5B**). Especially in study sites in Africa, where malaria transmission is high, those in the upsA group are more likely to be in non-1-to-1 DBLα-*var* relationships, relative to DBLα types in the non-upsA group. This observation was most prominent in Navrongo, with 12.44% of upsA DBLα types (123 of 989) showing a 1-to-many DBLα-*var* relationship compared to 0.97% of non-upsA DBLα types (110 of 11,358) with 1-to-many DBLα-*var* relationships (red, **Figure 5B**). Further classifications of these DBLα-*var* relationships according to the DBLα domain subclasses (**Figure S8** in **Data Sheet 1**) showed higher observed levels of DBLα1.4, DBLα1.7, and DBLα1.8 domain subclasses for upsA DBLα types in 1-to-many DBLα-*var* relationships in high-transmission study sites of Navrongo and Chikwawa.

Examination of *var* gene domain organizations showed the ‘NTS-DBLα-CIDRα-DBLδ-CIDRβ’ structure to be the most common across the lower levels of DBLα-*var* relationships (i.e., 1-to-1 to 1-to-5) (**Data Sheet 4**); this structure has been previously reported for *var* genes mainly in the non-upsA group (Kraemer and Smith, 2006; Rask et al., 2010). In contrast, there was greater variation in the domain organization of *var* genes associated with 1-to-many DBLα-*var* relationships, particularly in high-transmission study sites. For this latter category, in addition to the common ‘NTS-DBLα-CIDRα-DBLδ-CIDRβ’ structure, we also observed other *var* domain organizations in high frequencies (e.g., ‘NTS-DBLα-CIDRα-DBLβ-DBLγ-DBLδ-CIDRβ’ and ‘NTS-DBLα-CIDRα-DBLβ-DBLγ-DBLγ-DBLδ-CIDRβ’) (**Data Sheet 4**).

**Figure 6:**
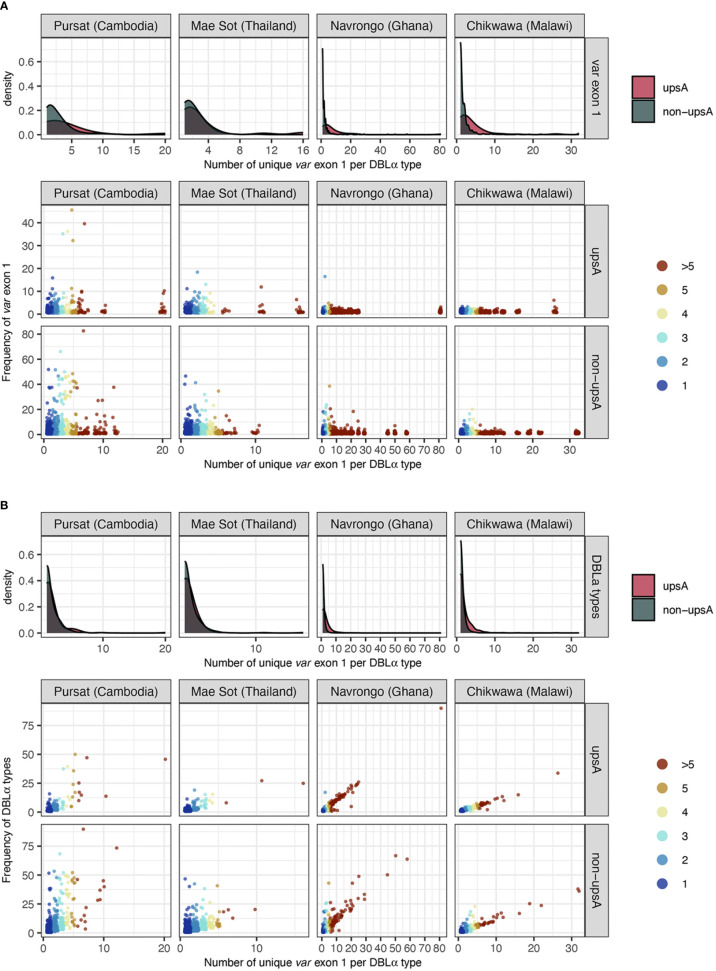
Frequency of **(A)** *var* exon 1 and **(B)** DBLα types (y-axes), categorised by DBLα-*var* relationships (x-axis). For each plot, density plots (top) show positively-skewed distributions of *var* exon 1/DBLα type sequences, indicating that most *var* exon 1/DBLα type sequences are involved in relatively specific DBLα-*var* relationships (i.e., 1-to-1, 1-to-2). Each point in the jitter plot (bottom) represents a unique *var* exon 1 or DBLα type sequence in a studied population, colored by its categorized DBLα-*var* relationship. For **Figure 6** **(B)**, although data points are expected to lie above the y=x boundary (i.e., if a DBLα type corresponds to at least n unique *var* exon 1 sequences, it must occur at least n times), it is possible for two different DBLα types to be aligned to the same *var* exon 1 due to the nature of centroid selection in the clustering step (see Rognes et al., 2016 for explanation on ‘centroid’). However, this observation is minimal (*var* exon 1 with >1 aligned DBLα types: 0.5%, 0.4%, 1.1%, and 0.4% for Pursat, Mae Sot, Navrongo, and Chikwawa, respectively).

Repeated observations of identical *var* exon 1 sequences that were categorized in 1-to-1 DBLα-*var* relationships in multiple isolates provide confidence that these inferences of specific relationships were likely true observations. Some of the *var* exon 1 sequences with these highly-specific 1-to-1 DBLα-*var* relationships were observed at high frequency within a study site and these were present in both upsA and non-upsA groups (**Figure 6A**). This was most prominent in sites with low malaria transmission, with some *var* exon 1 sequences that were categorized in 1-to-1 DBLα-*var* relationships seen frequently in Pursat (up to 52 times) and Mae Sot (up to 47 times). Some *var* exon 1 sequences in 1-to-1 DBLα-*var* relationships were also observed repeatedly in sites of high malaria transmission, but at relatively lower frequencies in Navrongo (up to 18 times) and Chikwawa (up to 8 times). These differences in frequencies between study sites with different malaria endemicities may reflect obvious differences in population frequencies, where parasite populations in low-transmission sites exhibit high linkage disequilibrium and clonality, features that are uncommon in high-transmission sites.

Time-specific analysis from alignments of DBLα types to *var* exon 1 from individual years within a study site (see Methods) showed even larger proportions of DBLα types with specific 1-to-1 DBLα-*var* relationships (**Figure S9** in **Data Sheet 1**). For instance, these 1-to-1 relationships were observed for an average of 88.4% of DBLα types per year (over five years) in Navrongo *vs* 74.2% of DBLα types in the previous site-specific (but not time-specific) analysis. The lack of deep longitudinal sampling of *var* sequences in the current dataset hinders further analyses of DBLα-*var* relationships over time, therefore we cannot rule out the possibility that these DBLα-*var* relationships will change or be affected by frequency-dependent selection (He et al., 2021) or short-term persistence of clones.

Unsurprisingly, the DBLα-*var* relationship was weak when relationships were explored in relation to *var* exon 1 sourced from a larger geographical region (i.e., country- or continent-specific *var* exon 1, see Methods), showcasing the underlying effects of spatial variation that are also seen with other molecular markers such as SNPs (Amambua-Ngwa et al., 2019). This was evident from substantially reduced proportions of DBLα types with 1-to-1 DBLα*-var* relationships and increased proportions of DBLα types with 1-to-many DBLα*-var* relationships within these larger spatial contexts (**Figure S10** in **Data Sheet 1**). This is expected given that, in the absence of gene flow (limited migration of people between distant locations and continents) and the prevailing view of typical mosquito dispersals of distances up to 5 to 25km (Costantini et al., 1996; Service, 1997), geographically-isolated parasite populations would undergo different recombination events in their *var* evolutionary histories and recombinants would have been generated from ‘parents’ or mosaic pieces that exist in a local *var* gene pool. This is also evidenced from local signatures found in recombinant sequences that are effective in informing geographic population structure (Tonkin-Hill et al., 2021). Thus, in this study, we show that the use of DBLα types to estimate *var* diversity is limited to the scale of a local site and caution should be applied when discussing the population genetics of DBLα types in relation to *var* in larger spatial contexts. As malaria control is local, this does not present a problem for the assessment of interventions and interactions with the theory of diversity thresholds (He and Pascual, 2021).

### 3.3 DBLα-*var* relationships are driven by DBLα type frequencies in high transmission

When determining DBLα-*var* relationships, DBLα types observed only once per population were, by default, assigned a 1-to-1 DBLα-*var* relationship. On the other hand, it is possible for more frequent DBLα types to be assigned to other DBLα-*var* relationships, the upper limit of these relationships being the frequency of the DBLα type in the studied population. Notably, the population frequencies of DBLα types in all study sites, regardless of transmission intensity, largely consisted of low-frequency DBLα types (**Figure 4D**) and this is concordant with observations of mostly rare DBLα types in other natural populations (Chen et al., 2011; Day et al., 2017; Ruybal-Pesántez et al., 2017; Ruybal-Pesántez et al., 2022).

Due to this attribute, the majority of DBLα types with 1-to-1 DBLα-*var* relationships were also DBLα types that have a frequency of one (**Figure 6B**). In study sites with low malaria transmission, we still observed many highly-frequent DBLα types that were still categorized in highly-specific DBLα-*var* relationships (e.g., 1-to-1, 1-to-2). In contrast, in study sites with high malaria transmission that also showcased relatively higher diversity levels of DBLα types and *var* exon 1 sequences, a strong correlation was found between DBLα type frequencies and DBLα-*var* relationships. Therefore, in high transmission, the more times a DBLα type was observed in the population, the higher the likelihood that the DBLα type was observed in many different *var* exon 1 in the same population, though notably, in these currently available datasets, the proportion of these high-frequency DBLα types in natural populations was relatively small (average of 1.4% of DBLα type in 1-to-many DBLα-*var* relationships across the four study sites). This also supports our earlier observation of greater proportions of upsA with 1-to-many DBLα-*var* relationships (**Figure 5B**), as DBLα types within the upsA group have been reported to be significantly more likely to be found in higher frequencies in parasite populations (Ruybal-Pesántez et al., 2017). The underlying forces driving this selection to conserve DBLα types through time remain to be explored.

### 3.4 Alignments of *var* exon 1 with the same DBLα type suggest findings of alleles and different genes

**Figure 7:**
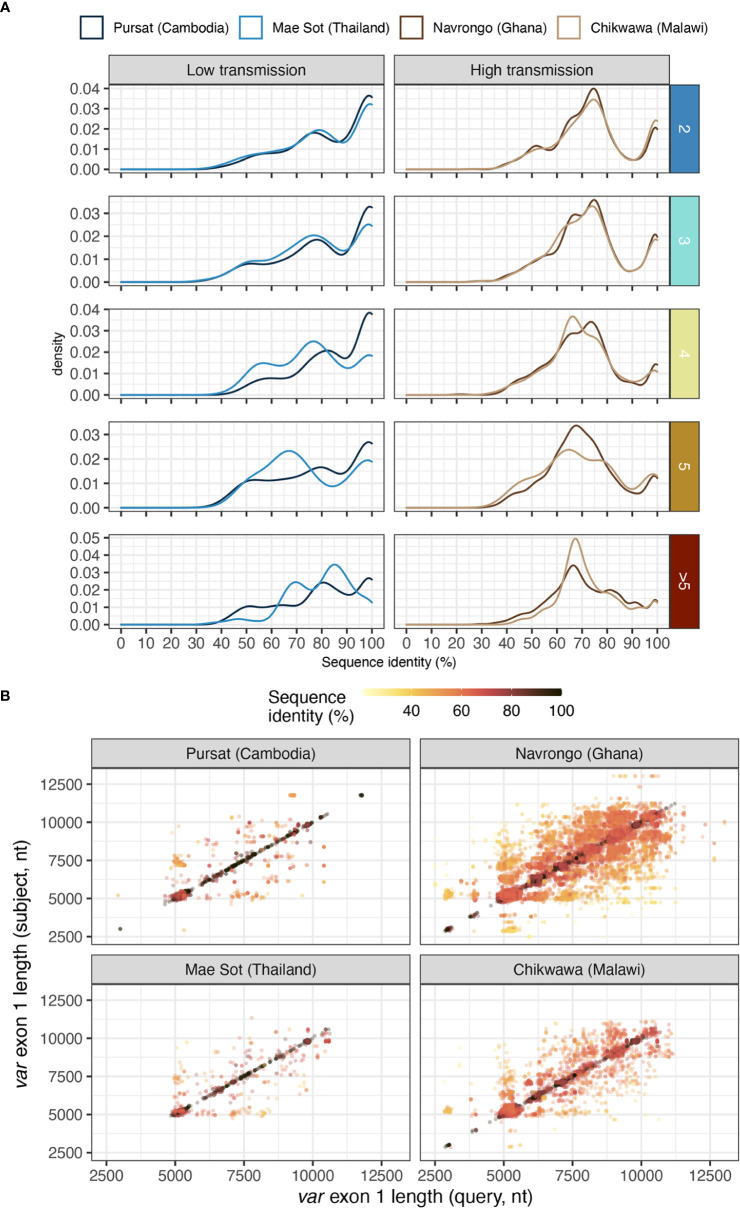
Sequence similarity of pairs of *var* exon 1 sharing a same DBLα type. **(A)** Distribution of nucleotide identities of pairs of aligned *var* exon 1. Horizontal rows represent the different levels of DBLα-*var* relationships (1-to-2, 1-to-3, …, 1-to-many). **(B)** Sequence length comparison (on x- and y- axes) for every pairwise aligned *var* exon 1 sequences that share the same DBLα type, coloured by sequence identity [i.e., nucleotide identity (%)].

Different *var* exon 1 sequences with the same DBLα type can display a range of sequence similarities, estimated from pairwise nucleotide identities from all *vs* all alignments of *var* exon 1 sequences within groups that share the same DBLα type (**Figure 7A**). In low malaria transmission sites (Pursat and Mae Sot), many of these *var* exon 1 sequences, especially those in 1-to-2 or 1-to-3 DBLα-*var* relationships, tended to share high sequence similarities (≥99%) with other *var* exon 1 of the same group (**Figure 7A**). In contrast, in Navrongo and Chikwawa, while there was a substantial portion of *var* exon 1 that also share high levels of sequence similarity (≥99%), there was an even greater portion of *var* exon 1 sequences that are dissimilar, mostly sharing approximately 60 to 80% of nucleotide identity (**Figure 7A**), and these were observed for sequences classified in both upsA and non-upsA groups (**Figure S11** in **Data Sheet 1**).

Notably, because *var* sequences were highly variable in length, the aforementioned sequence identity between two aligned *var* exon 1 sequences was calculated over the length of an alignment (including terminal gaps). Therefore, pairs of aligned *var* exon 1 sequences with high sequence similarities (e.g., ≥99%) were both highly similar in sequence and length. In contrast, *var* exon 1 pairs that exhibited low sequence similarity (e.g., <80%) can represent genes that differ in sequence or in length, or both, suggesting that these sequences were likely very different *var* genes (**Figure 7B**). Upon detailed examination of several randomly-selected alignments within each range of sequence identities (**Figure S12** in **Data Sheet 1**), alignments of *var* exon 1 pairs with ≥99% sequence identity showed largely-conserved sequence regions. On the other hand, for alignments with low sequence similarity, conserved regions were observed on the 5’ end of alignments and were truncated, potentially indicative of a putative recombination breakpoint in these *var* exon 1 sequences.

The evolution of *var* genes by ectopic recombination makes it difficult to define an allele of any *var* gene even in a population, especially in populations with high malaria transmission as seen in Ghana and Malawi. In this study, DBLα-*var* relationships were determined conservatively; i.e., two *var* genes in a group that share a same DBLα type were considered to be different genes even if there was only a single polymorphism found outside of the DBLα tag region, leading to an inference of a 1-to-2 DBLα-*var* relationship. If we could properly define *var* gene alleles, we would expect to observe even higher proportions of DBLα types with highly-specific 1-to-1 DBLα-*var* relationships, particularly in sites with low malaria transmission.

To check whether *var* genes that shared the same DBLα type also shared the same or different domain organizations, we further compared the domain organization of *var* exon 1 sequences in the simplest category of 1-to-2 DBLα-*var* relationships in three bins of nucleotide identity (i.e., in ranges from low to high identities of 60-80%, 80-99%, and ≥99%) (**Data Sheet 4**). For alignments reporting relatively higher sequence similarities, an average of 98.4% and 82.8% of alignments within the ≥99% and 80-99% categories, respectively, were observed to be between two *var* genes with identical domain organizations, majority of which have the common ‘NTS-DBLα-CIDRα-DBLδ-CIDRβ’ domain organization. In contrast, this characteristic was observed for an average of 47.1% of alignments in the 60-80% sequence identity category. For this category, there were a number of alignments between two *var* genes with different domain compositions and organizations. Most notably, in all study sites, a relatively large proportion of aligned *var* genes (average 20.5% of alignments) consisted of cases where one of the genes has the ‘NTS-DBLα-CIDRα-DBLδ-CIDRβ’ structure and the other gene has a ‘NTS-DBLα-CIDRα-DBLδ-CIDRγ’ structure; structurally, these *var* genes differ in only the last CIDRβ/γ domain. Whether these alignment breakpoints between two different domain structures lie intra- and/or inter-domain (and in which domain) is of interest and remains to be explored in future comprehensive analyses.

## 4 Discussion

Here, we present analysis of population data showing largely 1-to-1 DBLα-*var* relationships in multiple local sites of varying transmission intensities. This study provides *in vivo* evidence to question the rates of mitotic recombination reported *in vitro* (Claessens et al., 2014) as such recombination should lead to a higher frequency of 1-to-many relationships. Alternatively, these recombinants may be created but are selected against. Overall, our findings justify the use of DBLα tags as a reasonable surrogate for *var* diversity within and between hosts. It also supports the translation of these findings to use DBLα tags for *var* surveillance.

DBLα-*var* relationships appear to be largely determined by the frequencies of DBLα types in a local population; i.e., greater proportions of rare DBLα types result in majority of DBLα-*var* relationships being highly specific. This is especially the case in high malaria transmission settings, which are often characterized by a large proportion of DBLα types occurring at very low frequencies, particularly in the non-upsA group (Ruybal-Pesántez et al., 2017). This observation, coupled with the predominantly non-overlapping structure of DBLα types in high transmission, consequently underpins a specific application that has been proposed (Labbé et al., 2023; Ruybal-Pesántez et al., 2022; Tiedje et al., 2022), which is to measure complexity/multiplicity of infection (MOI) from the number of non-upsA DBLα types identified in an isolate (i.e., infected individual). Based on the assumption that there are approximately 45 unique non-upsA *var* genes in a parasite genome (Ruybal-Pesántez et al., 2022), the current approach has been to estimate the number of diverse parasite genomes in an isolate by counting the number of sets of 45 unique non-upsA DBLα types. The finding of predominantly 1-to-1 relationships for non-upsA DBLα types in high transmission strengthens the strategic assumption that counts of unique non-upsA DBLα types are representative of actual counts of unique *var* genes in an isolate. This approach presents an alternative for estimating MOI in highly-multiclonal isolates (i.e., MOI > 5), for which current methods using SNP and microsatellite data are limited (Chang et al., 2017; Gerlovina et al., 2022).

Strong DBLα-*var* relationships, especially those observed in areas with high malaria transmission, extends our ability to estimate diversity of not only DBLα tags but of *var* genes. For example, previous work by Tiedje et al. (2022) reported the decline in observed DBLα type diversity in relation to an indoor residual spraying (IRS) intervention over 3 years that reduced transmission intensity by >90% and parasite prevalence by 40-50%. Notably, this is done by counting the number of different DBLα types detected to obtain an estimate of the minimum number of different *var* genes in a population. The finding of a proportion of DBLα types with non-1-to-1 DBLα-*var* relationships, however, indicates that using DBLα types to estimate local population size (Barton, 2010) or diversity thresholds (He and Pascual, 2021) of adaptive *var* genes will present a degree of underestimation. While the currently-available *var* and DBLα datasets are under-sampled, especially in high-transmission settings, we hypothesize that the pattern of DBLα-*var* relationships will remain consistent with greater sampling, given the trend of uncovering largely rare DBLα types in non-overlapping repertoires in high malaria transmission (Ruybal-Pesántez et al., 2017) and especially if we can properly define alleles. In the absence of deep site-specific sequencing and assembly of *var* genes, there is also an avenue for a future method to be developed to better distinguish alleles and to quantify this underestimation, taking into account DBLα-*var* relationships (e.g., 1-to-1, 1-to-2, 1-to-many) in a natural population, much of which are correlated with DBLα type frequencies.

In addition to estimating *var* diversity using DBLα, previous studies have shown that DBLα tags contain signatures of geographic variation within the African continent. This allows one to track DBLα types to specific localities, countries and continents when analyzed using a jumping hidden Markov model (Tonkin-Hill et al., 2021) with greater resolution than has been reported for large numbers of SNPs. Therefore, with regard to malaria surveillance, with this application termed ‘*var*coding’ (i.e., one single PCR to recover DBLα tags in an isolate), we can obtain information about geographic origin of parasites, MOI as well as estimate *var* gene diversity. This *var* coding methodology using DBLα tag sequences is akin to microhaplotyping (LaVerriere et al., 2022; Tessema et al., 2022) and is potentially more informative, especially in high transmission.

Even with recent advancements in genomics methods and technologies, there remains a dearth of available genomic resources for *var* genes. The exception has been the impressive efforts of Otto et al. (2019), upon whose work we have based this analysis. Understandably, the *var* system is so diverse and complex that obtaining large comprehensive collections of *var* genomic sequences will, for now, continue to be costly and will require a global collaborative effort. To this end, the justified ability to now use DBLα tags in genomic surveillance of *var* diversity and MOI estimation is highly valuable, especially in high-transmission settings, where malaria continues to pose a major threat to public health.

## Data availability statement

Publicly available datasets were analyzed in this study. The names of the repository/repositories and accession number(s) can be found in the article/**Supplementary Material**.

## Author contributions

KPD conceptualized the research idea and project. MT and KPD designed the research and direction of analyses. MT performed formal analysis of the data and wrote the initial draft of the manuscript. KPD, YC, and HS critically reviewed and edited the manuscript. All authors read and approved the final version of the manuscript.
