# Supplementary material for: Unravelling var complexity: Relationship between DBLα types and var genes in Plasmodium falciparum
Source: Front Parasitol. 2023 Jan 9;1:1006341. doi: 10.3389/fpara.2022.1006341 (PMC10060044; doi:10.3389/fpara.2022.1006341)
Supplement: Supplementary file 1 [file DataSheet_1.pdf]

frontiers

Supplementary Material

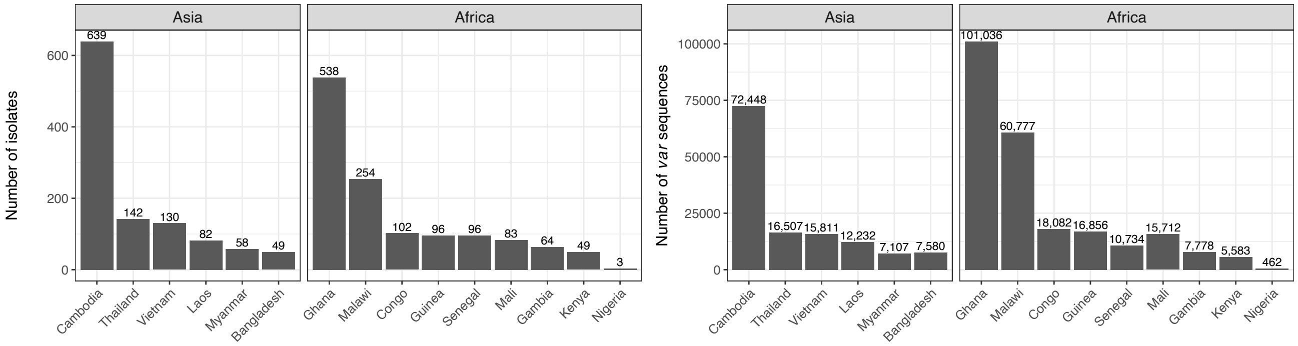

Figure S1. Number of isolates **(left)** and *var* sequences **(right)** per country, from the 'Full Dataset' of assembled *var* published by Otto et al. (2019).

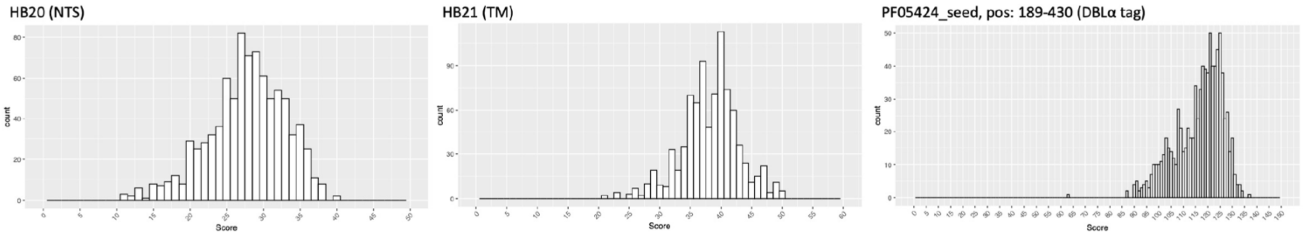

Figure S2. Distribution of domain scores from *hmmsearch* alignments of *var* or DBLα tags of sequences from 16 whole genome assemblies (downloaded from PlasmoDB (Otto et al. (2018) and NCBI) to homology blocks (HB20 and HB21) or to PF05424 domain alignment.

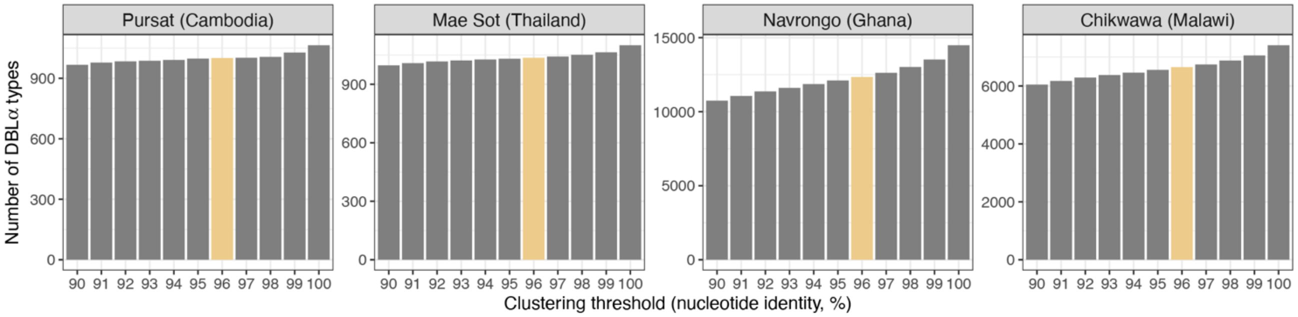

Figure S3. Clustering of DBLα tags into DBLα types over a range of thresholds (90-100% nucleotide identity). A 96% nucleotide identity threshold (yellow) was used to generate DBLα types analyzed in this study.

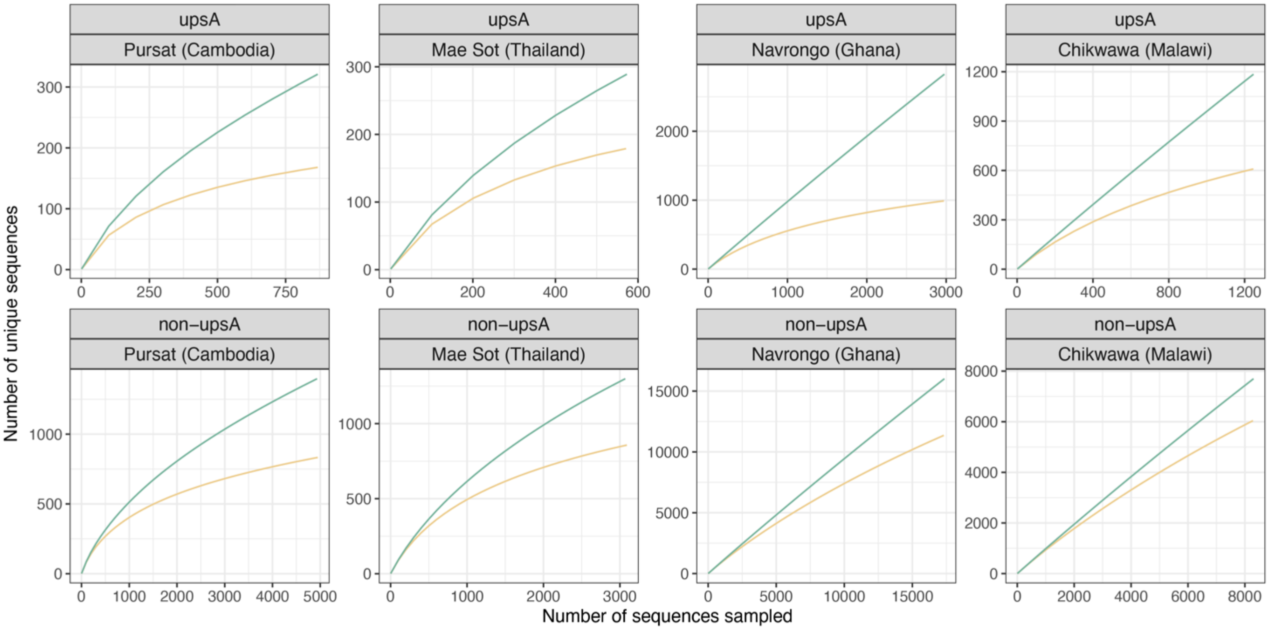

Figure S4. Sampling curves of *var* exon 1 and DBLα type sequences by ups group.

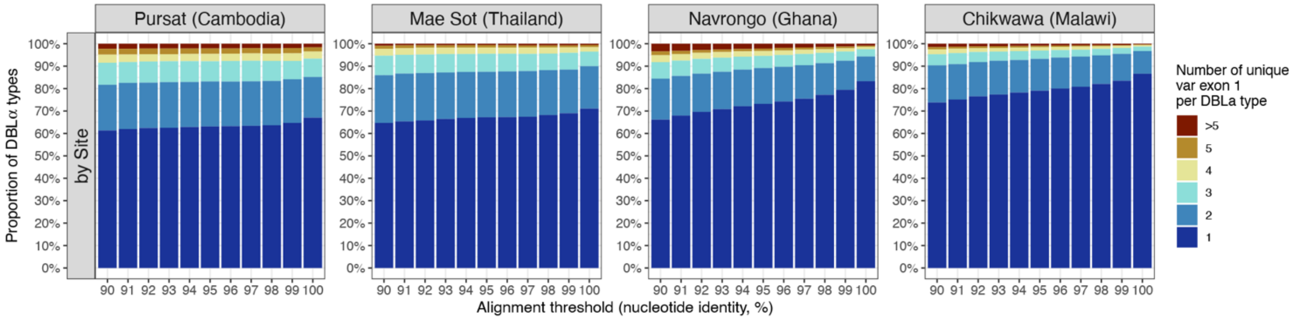

Figure S5. DBLα-*var* relationships based on alignments of DBLα types to *var* exon 1. The relationship between a DBLα type and *var* exon 1 sequences is represented by the number of unique *var* exon 1 sequences a DBLα type aligns to, at clustering and alignment thresholds ranging from 90 to 100% nucleotide identity.

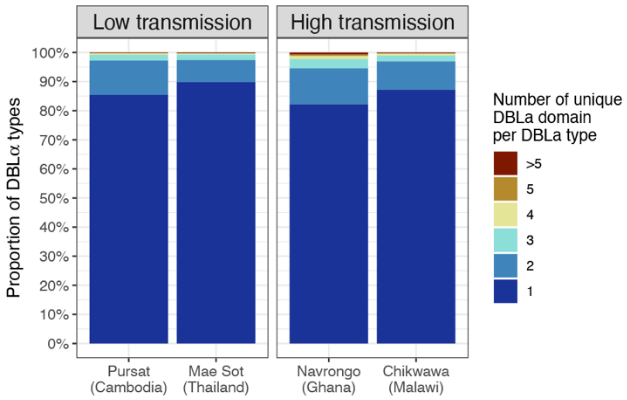

Figure S6. DBLα type-domain relationships based on alignments of DBLα types to sequences encoding the complete DBLα domains. The relationship between a DBLα type and sequences encoding the complete DBLα domain is represented by the number of unique sequences encoding the complete DBLα domain that share a same DBLα type (clustered at 96% nucleotide identity threshold).

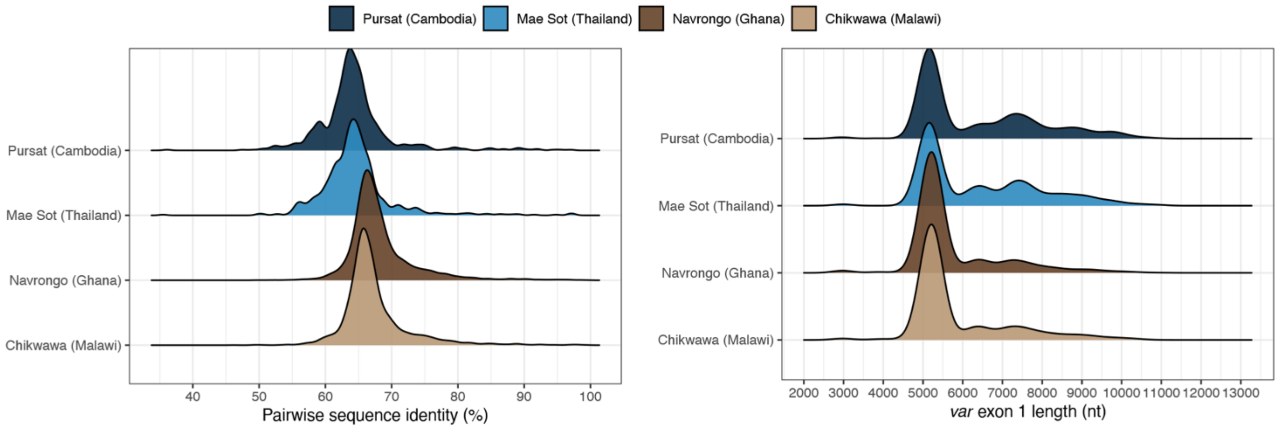

Figure S7. Distribution of sequence similarities **(left)** and lengths **(right)** of *var* exon 1 sequences categorized in 1-to-1 DBLα-*var* relationships.

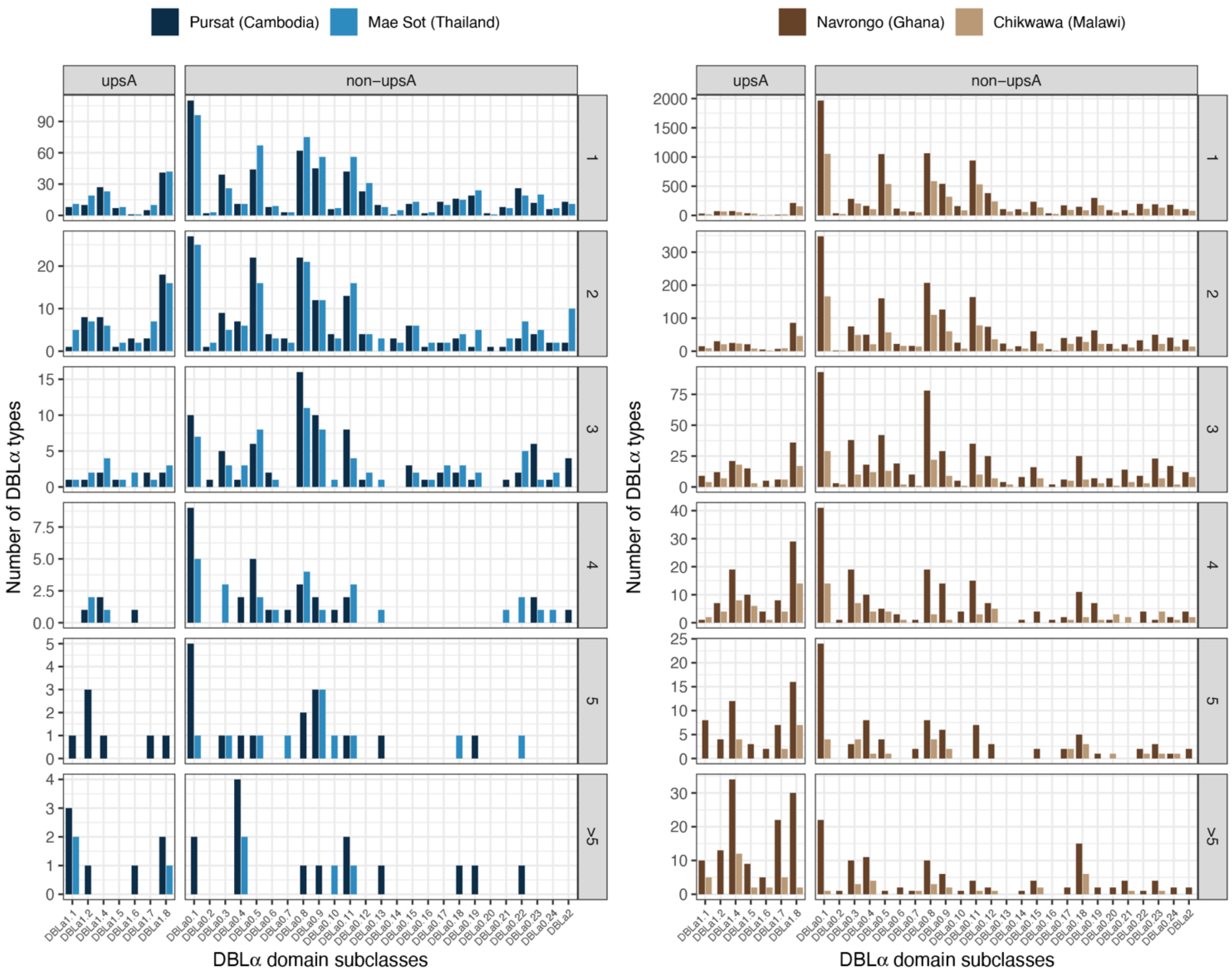

Figure S8. Distribution of DBLα types according to DBLα domain subclasses. Horizontal rows represent the different levels of DBLα-*var* relationships (1-to-2, 1-to-3, …, 1-to-many).

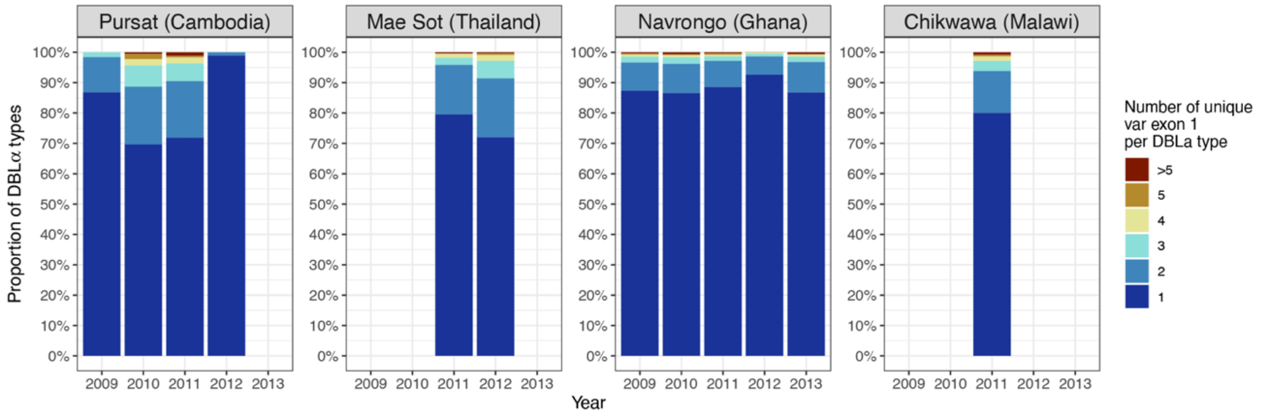

Figure S9. DBLα-*var* relationships based on alignments of DBLα types to time-specific *var* exon 1. The relationship between a DBLα type and *var* exon 1 is represented by the number of unique *var* exon 1 sequences that share a same DBLα type (clustered at 96% nucleotide identity threshold).

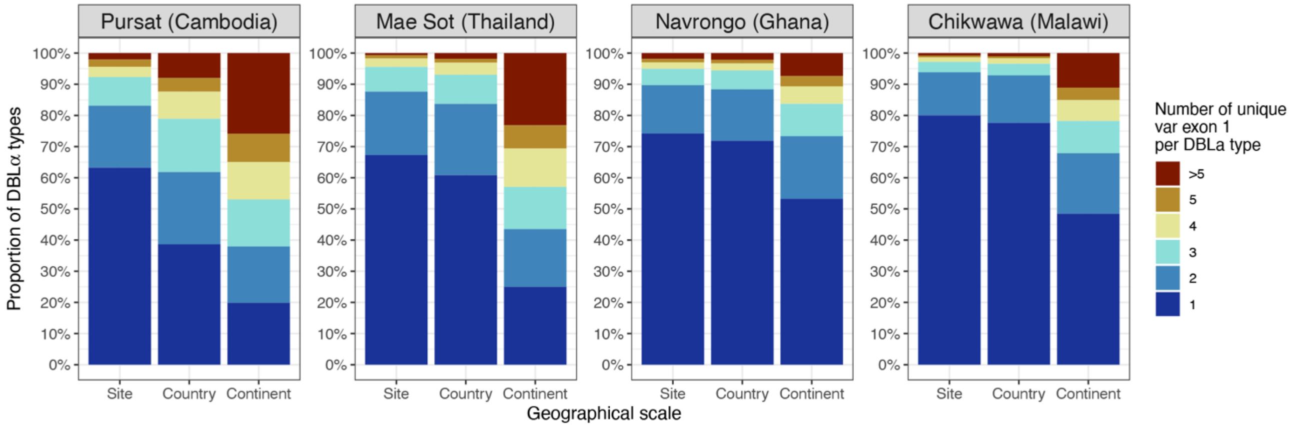

Figure S10. DBLα-*var* relationships based on alignments of DBLα types to site-, country- and continent-specific *var* exon 1. The relationship between a DBLα type and *var* exon 1 is represented by the number of unique *var* exon 1 sequences that share a same DBLα type (clustered at 96% nucleotide identity threshold).

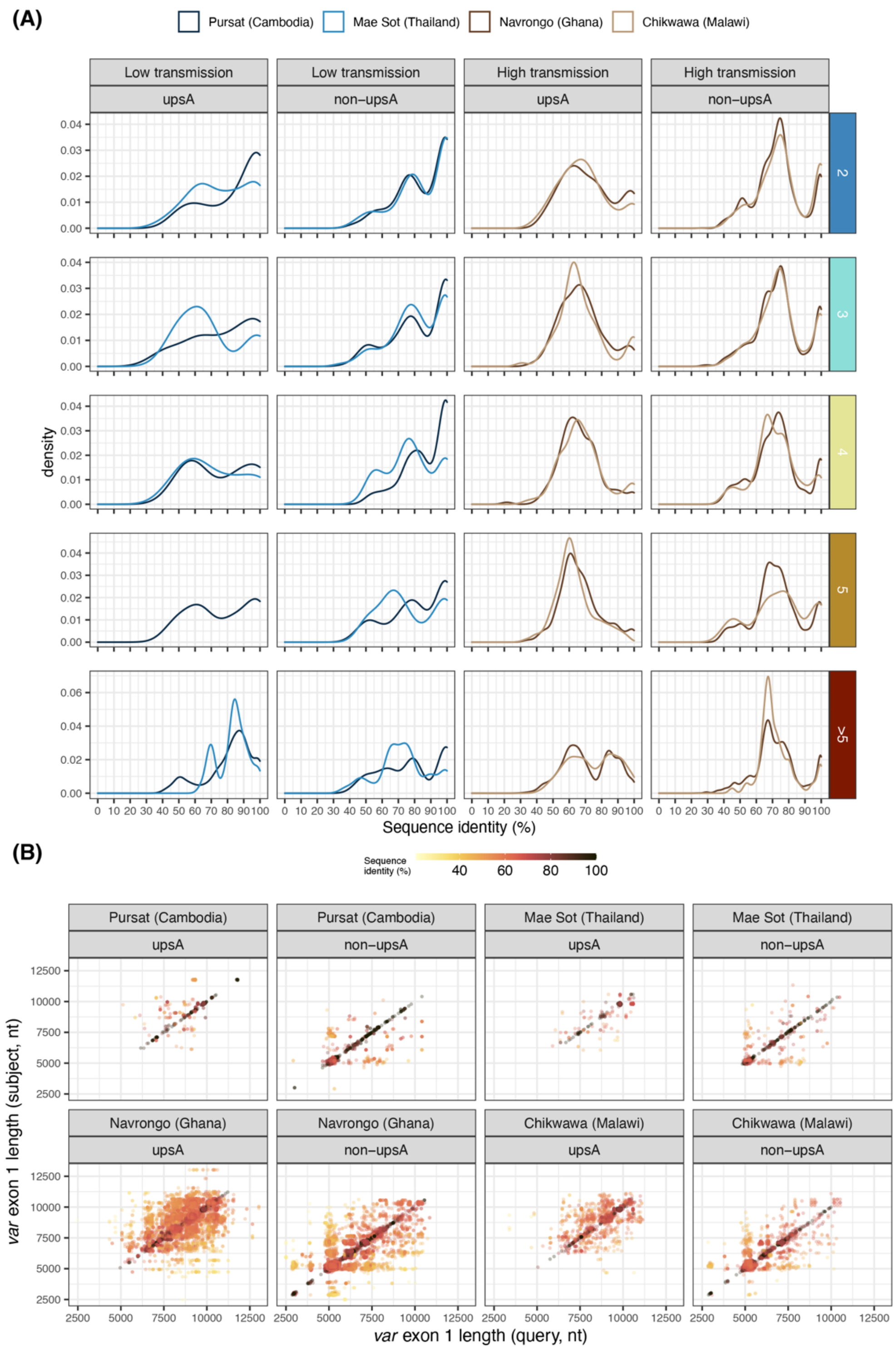

Figure S11. Sequence similarity of pairs of *var* exon 1 sharing a same DBLα type, segregated by ups groups (upsA and non-upsA). **(A)** Distribution of nucleotide identities of pairs of aligned *var* exon 1. Horizontal rows represent the different levels of DBLα-*var* relationships (1-to-2, 1-to-3, …, 1-to-many). **(B)** Sequence length comparison (on x- and y- axes) for every pairwise aligned *var* exon 1 sequences that share the same DBLα type, colored by sequence identity (i.e., nucleotide identity (%)).

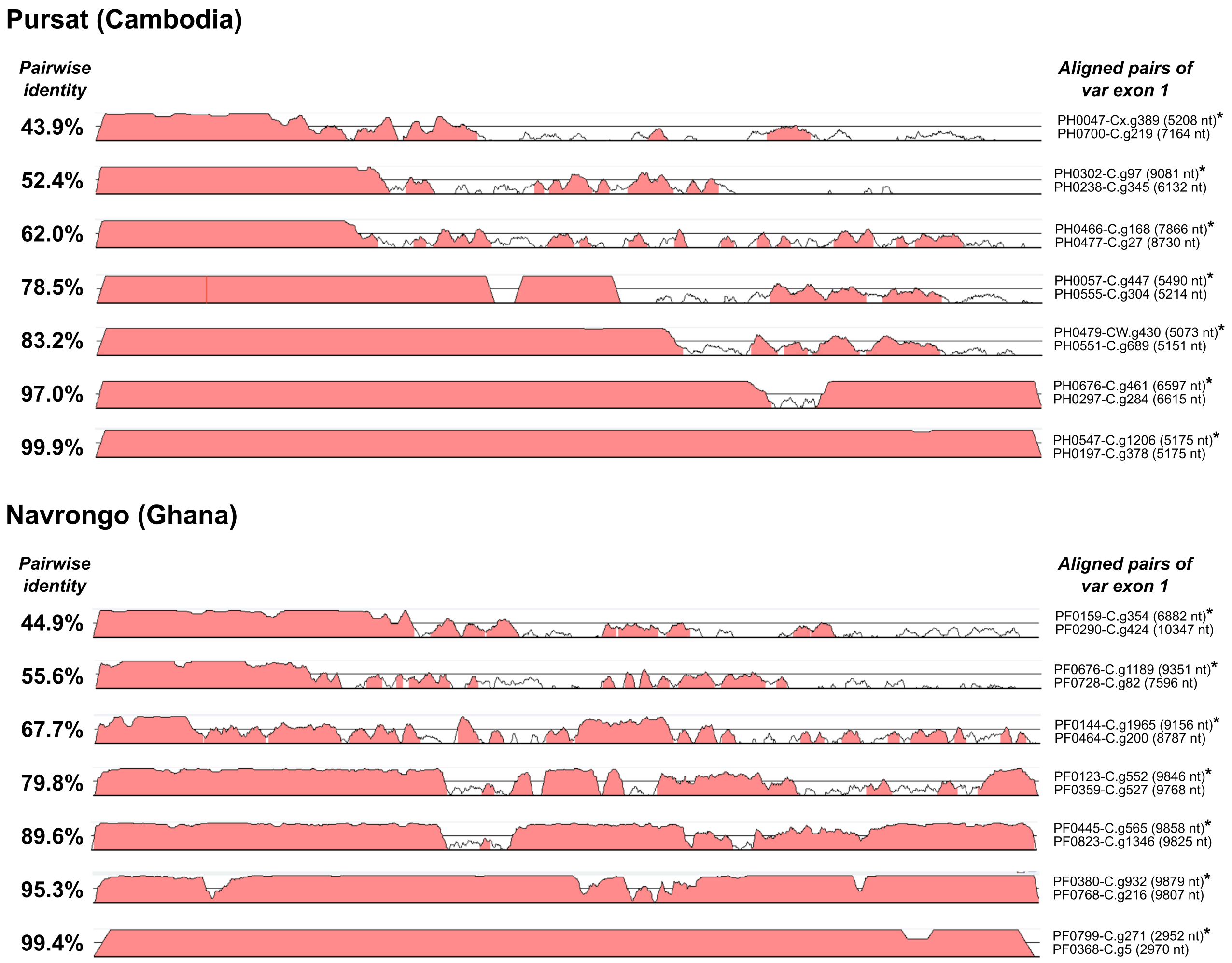

Figure S12. Visualized examples of pairwise alignments of *var* exon 1 that share a same DBLα type. **(Left)** Pairwise nucleotide identity reported from *vsearch* alignment. **(Center)** Pink regions indicate conserved sequence regions that share a minimum of 70% identity. **(Right)** Sequence IDs and lengths of aligned *var* exon 1, "*" represents sequence on each x-axis.
